# Buserelin shortens the estrus-to-ovulation interval and improves the pregnancy outcomes in gilts treated with a fixed-time artificial insemination

**DOI:** 10.3389/fvets.2025.1612713

**Published:** 2025-06-18

**Authors:** Zhicheng Shi, Zongyu Wang, Lei An, Zhi Li, Linghua Cheng, Yuan Yue, Min Guo, Xiaodong Wang, Haiqing Liu, Li Ren, Jianhui Tian, Qin Li, Shumin Wang

**Affiliations:** ^1^Frontiers Science Center for Molecular Design Breeding of the Ministry of Education, China Agricultural University, Beijing, China; ^2^Key Laboratory of Animal Genetics, Breeding and Reproduction of the Ministry of Agriculture and Rural Affairs, China Agricultural University, Beijing, China; ^3^State Key Laboratory of Animal Biotech Breeding, China Agricultural University, Beijing, China; ^4^National Engineering Laboratory for Animal Breeding, China Agricultural University, Beijing, China; ^5^College of Animal Science and Technology, China Agricultural University, Beijing, China; ^6^Beijing Great Wall Danyu Livestock Products Co., Ltd., Beijing, China

**Keywords:** buserelin, gonadorelin, the estrus-to-ovulation interval, gilts, fixed-time artificial insemination

## Abstract

Fixed-time artificial insemination (FTAI) is currently a standardized protocol for pig reproductive management. Efficient ovulation synchronization induced by GnRH analogs is critical for ensuring the pregnancy outcomes of FTAI. However, among the widely used GnRH analogs, the degree of synchronization and timing of ovulation remain unclear in gilts. In the present study, we focused on the estrus-to-ovulation interval, a key component in fertility management programs, and directly compared the follicular dynamics and timing of ovulation, and the subsequent pregnancy outcomes between two well-established GnRH analogs buserelin and gonadorelin. 224 prepubertal Large White gilts, randomly divided into three independent batches, were allotted to this study. The administration of PMSG was aligned with the FTAI protocol, with gonadorelin or buserelin injections administered upon detection of estrus onset in gilts. Ovarian ultrasonography was performed at the onset of estrus. Serum samples were collected for LH detection. Total piglets born, born alive, and other performance indicators were measured. Our results showed that buserelin-treated gilts exhibited an earlier ovulation, as well as a shorter estrus-to-ovulation interval and a centralized ovulation duration, with 81.5% ovulation occurring within 24–48 h after buserelin injection. Additionally, although the pregnancy rate and farrowing rate didn't differ between the two analogs, buserelin administration is beneficial for the number of total piglets and the piglet index. In conclusion, our data demonstrate that buserelin has advantages in centralizing induced ovulation and thus improving FTAI outcomes.

## 1 Introduction

Exogenous gonadotropin-controlled ovarian stimulation promotes animal reproductive management. In particular, it facilitates synchronous estrus, synchronizes ovulation and stimulates multiple ovulation in pigs (1, 2), which enables fixed-time artificial insemination (FTAI) and “All-In All-Out” swine reproduction system (3). The estrus-to-ovulation interval is essential for estimating the best time for FTAI because it reflects the degree of synchronization and timing of ovulation; thus, it is thought of as a key indicator that influences the outcomes of FTAI (4, 5). It has been reported that a shorter estrus-to-ovulation interval is strongly associated with favorable farrowing rate and pregnancy outcomes. Insemination within the optimal interval is correlated positively with higher pregnancy outcomes (6). By contrast, gilts with longer estrus-to-ovulation interval may miss the optimal time window for insemination. Additionally, conducting insemination outside the optimal interval also results in lower rate of conception and poor pregnancy outcomes (7).

Gonadotropin-releasing hormone (GnRH) analogs can mimic endogenous GnRH to stimulate the synthesis and secretion of the gonadotropic hormones, i.e., follicle stimulating hormone (FSH) and luteinizing hormone (LH). Thus, GnRH analogs have been widely used as ovulation-inducing drugs in the reproductive management of gilts and sows (8), in particular for triggering an endogenous surge of LH, thereby synchronizing ovulation during the FTAI procedure (9, 10). A variety of GnRH analogs have been developed for ovulation induction after estrus synchronization by altrenogest in gilts or weaned multiparous sows to achieve an additional synchronization effect that can be used to fulfill FTAI protocols (1, 11–14). Currently, gonadorelin administration has been widely used as a standardized protocol for ovulation induction in the FTAI procedure (15–18). However, several evidence suggested that gonadorelin injection may be associated with of more dispersed ovulation, as revealed by a longer duration of the ovulation in comparison with buserelin (8, 15, 19). Buserelin, a synthetic gonadotropin-releasing hormone (GnRH) analog, has been extensively utilized in modern livestock production systems for swine, cattle, and sheep and showed its multifunctional biological efficacy in enhancing the litter size of gilts (20), strengthening the testicular function in rams (21), and promoting progesterone synthesis pathway during the early luteal phase in sheep (22). Moreover, Buserelin has been reported to have the function of increasing the concentration of FSH and LH which may promote the development of follicles in cows (23) and shortening of calving to conception interval in cows (24). Studies have documented its biological efficacy in narrowing the estrus-to-ovulation interval in weaned sows (25). However, the direct comparison under well-controlled conditions, including randomized gilt assignments and a standardized FTAI protocol, remains lacking. The follicular dynamics and timing of ovulation, as well as the subsequent pregnancy outcomes, have not been compared directly between buserelin and gonadorelin to ensure rigorous experimental comparability and minimize confounding variables.

Gilts are the foundation of efficient breeding herd (26), and efficient and timely ovulation induction is basis for the reproductive performance of gilts inseminated using FTAI protocol. Systematic screening of ovulation-inducing analogs with optimal efficacy therefore represents a critical pathway for refining present FTAI methodologies. Furthermore, achieving tightly clustered ovulation patterns through controlled hormonal regulation establishes essential physiological prerequisites for maintaining satisfactory conception rates following single fixed-time insemination protocols (12, 27).

Herein, by implementing high-frequency follicular monitoring technology, we tested the degree of synchronous ovulation in gilts induced by the two GnRH analogs. We also compared pregnancy outcomes after buserelin and gonadorelin administration, to evaluate the efficacy of the two GnRH analogs in supporting FTAI protocols of gilts. The results can serve as a valuable foundation for guiding protocol optimization of FTAI and single-dose insemination strategies in both gilts and sows.

## 2 Materials and methods

The study was conducted on a farm of the Beijing Breeding Center in 2023 under protocols approved by the China Agricultural University Institutional Animal Care and Use Committee. Gilts were in good health during the course of the study.

### 2.1 Animal treatment and experimental design

Prepubertal Large White gilts (*n* = 224), weighing 140 kg ± 5 kg, aged 220 to 230 days, were allotted in this study. After two natural estrous cycles, gilts were housed individually in an environmentally controlled room maintained at approximately 22 ± 1°C, and the humidity at 60.0–70.0%. The feeding system for gilts consists of stainless steel feeders, allowing them to eat freely, and nipple waterers that provide unrestricted access to water. Gilts were maintained on a standard diet identical to that utilized in prior investigations. Main ingredients, chemical composition, and nutritional value of the diet were showed in Table 1.

**Table 1:** Ingredients, chemical composition, and nutritional value of the experimental diets for the pigs (as-fed basis).

**Item**	**Ingredient (g/kg)**
Corn	580
Soybean meal	193
Rice bran meal	100
Wheat bran	81
Dicalcium phosphate	10.2
Limestone	11.5
Salt	4.1
L-Lysine.	2
DL-Methionine	0.6
L-Threonine	0.6
Soybean oil	8
Choline chloride	1
Vitamin and mineral premix (18)	8
Chemical composition	
NE (kcal/kg)	2,180
EE (%)	3.7
CF	4.3

NE, net energy; EE, ether extract; CF, crude fibre.

**Figure 1:**
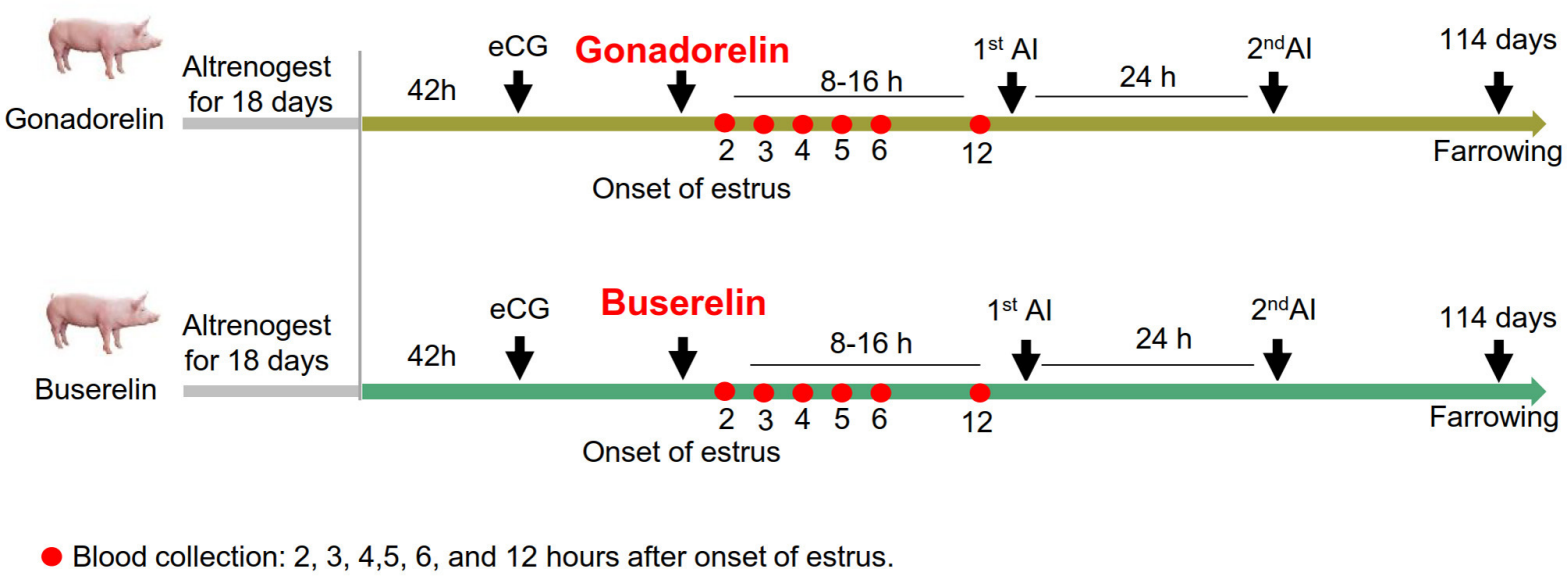
FTAI protocols using gonadorelin or busherelin-induced ovulation synchronization. Gilts undergoes an 18-day altrenogest-based estrus synchronization followed by follicular stimulation with eCG. At the onset of estrus, gilts receive GnRH analogs (gonadorelin or buserelin). Subsequently, the first artificial insemination is performed 8–12 h post-estrus onset, followed by a second insemination at a 24-h interval. Besides, high-frequency blood sampling at critical time points (2, 3, 4, 5, 6, and 12 h post-estrus onset), marked with red dots, were taken for maternal luteinizing hormone (LH) dynamics characteristic. Consective follicular ultrasonography was employed to track follicular development and ovulation from estrus to ovulatory phases.

**Figure 2:**
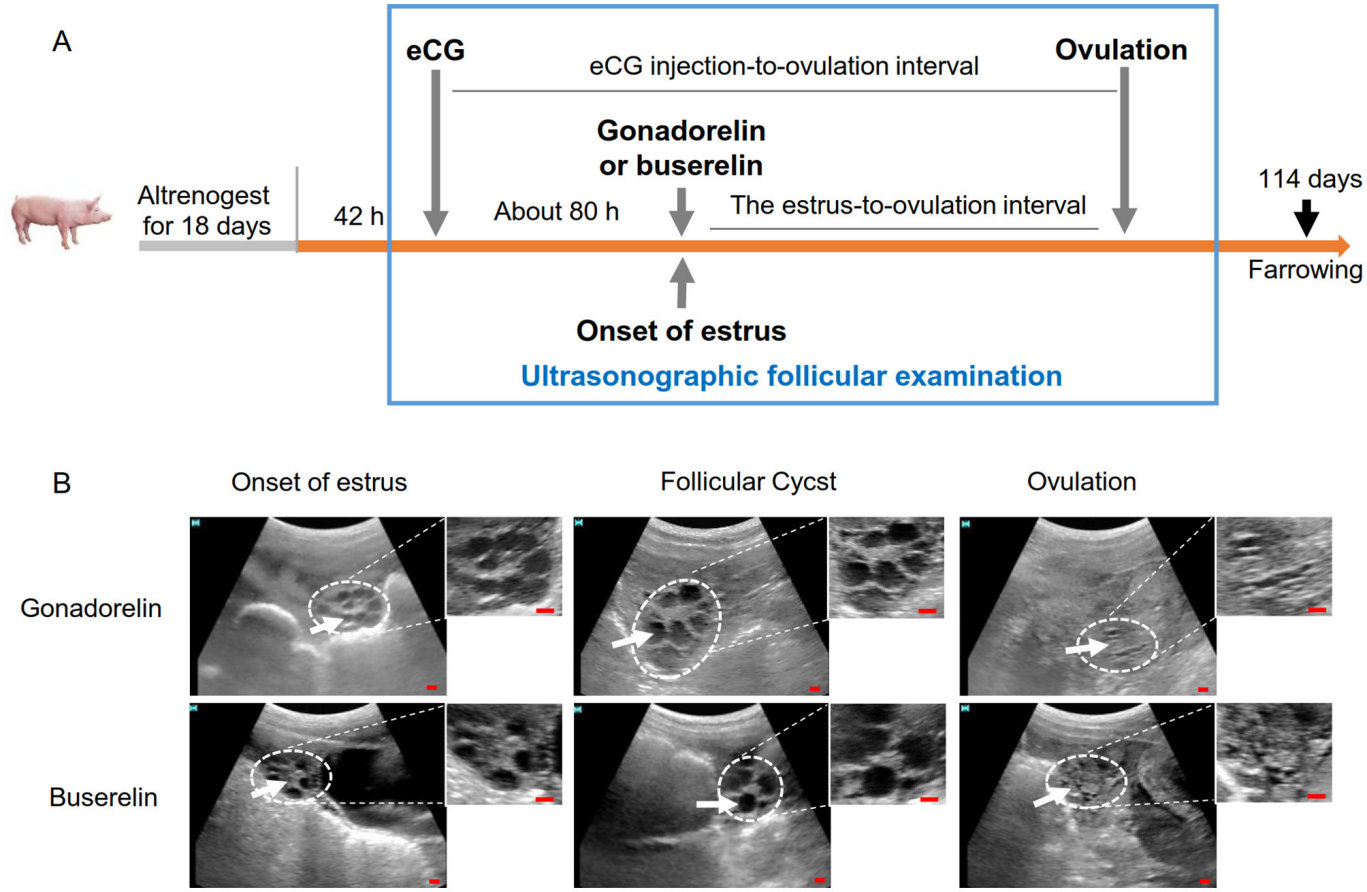
The ovarian ultrasonography examination workflow and the typical images of follicles in gilts. **(A)** Schematic diagram of the experimental workflow. High-frequency follicle monitoring was carried out from estrus to ovulatory phases, labeled with a blue rectangular box. **(B)** Photographs of the porcine follicular ultrasound. Ultrasound images were captured by HS-1600V device. Ovaries appeared as gray or white echoes (white dashed lines). Onset of estrus, follicles were visualized as dark circular shapes (white arrows) and during the post-ovulation stage, the dark clusters within the ovarian stroma were regarded as immature antral follicles. Scale bars: 10 mm.

The experimental design was illustrated in Figure 1. In this study, gilts were maintained under identical controlled conditions, including randomized gilt assignments and a standardized fixed-time artificial insemination (FTAI) protocol, to ensure rigorous experimental comparability and minimize confounding variables. Besides, the present study was conducted through three independent trials. Briefly, gilts were randomly allotted to two treatments, including gonadorelin group (*n* = 120) and buserelin group (*n* = 104). Gilts of the two groups were administered 5 mL of glucose syrup via a feeding gun 3 days prior to the altrenogest administration for habitual feeding. Subsequently, gilts were orally administered altrenogest (Ningbo Sansheng, Zhejiang, China), (20 mg, 15:00) daily for 18 days. After that, a dose of 1,000 IU equine chorionic gonadotropin (eCG), (Ningbo Sansheng, Zhejiang, China) was injected intramuscularly 42 h after the final altrenogest administration. Then, daily estrus monitoring was conducted on several days after the altrenogest withdrawl and male pigs were exposed right ahead of the female gilts for estrus detection two times a day. The majority of gilts exhibited a standing reflex approximately 80 h post-injection of eCG (Figure 2A), and no difference can be detected between these two groups indicating the randomness of the group assignment. The FTAI protocol was performed following established methodologies (15, 28, 29). Typically, gilts in estrus were immediately injected intramuscularly with 100 μg (2 mL) gonadorelin (Ningbo Sansheng, Zhejiang, China) or 10 μg (2.5 mL) buserelin (Ningbo Sansheng, Zhejiang, China). And then the artificial insemination (AI) procedures remained consistent across the two groups. Specifically, the first insemination was performed 8–12 h after gonadorelin or buserelin injection, with the second insemination conducted 24 h later. Semen of Large White boar were obtained from the core breeding center of Beijing Great Wall Danyu Livestock Products Co., Ltd. Semen were collected and evaluated according to standardized protocols (30). In the present study, 60 mL semen was used, containing a sperm count of 2 × 10^9^ per dose, with over 85.0% motility rate.

### 2.2 Estrus detection

Following altrenogest withdrawal, estrus detection was performed twice a day at 9:00 and 15:00, respectively (31), using fence-line boar contact with the backpressure test. Briefly, estrus detection was conducted through concurrent boar exposure (olfactory stimulation) and application of manual lumbosacral pressure, with standing reflex serving as the definitive indicator of estrus onset (32). Additionally, the altrenogest withdrawal to estrus interval was recorded for each gilt. Meanwhile, the estrus rate of each group was analyzed.

### 2.3 Monitoring of follicular dynamics and timing of ovulation

Ovarian ultrasonography was performed with a portable ultrasound machine, HS-1600V device with 5.0-MHz sectorial probe (Honda Electronics, Japan), as previously described (33). The parameters of HS-1600V ultrasound device used in the present study are as follows: frequency (F), 5.0MHz; gain (A), 100; grayscale (G), 95; depth (D), 95 and resolution (R), 110. When examined via ultrasound, follicular fluid exhibited minimal reflection, resulting in a dark hypoechoic appearance, whereas solid structures demonstrated significant reflection, appearing as gray or white echoes (Figure 2B). Follicular diameter was quantified by calculating the mean of the five largest follicles observed within the field of view at each time point, ensuring standardized measurement consistency across experimental replicates. Follicular development and ovulation were assessed according to established protocols (15, 33). During scanning, gilts were adequately restrained and immobilized with minimal stress. The diameter of gilt large follicles is about 5–6.5 mm, and follicles reach diameters ~6.0 mm during estrus. Ovulation timing was determined via serial ovarian ultrasound examinations at 8-h intervals. Typically, a significant reduction in large follicle count (follicles ≥6 mm while the number of follicles ≤ 2) during subsequent examinations marked the initial observation time as the ovulation point (29). Follicular cysts were defined as a pathological condition characterized by abnormally enlarged follicles (>8 mm diameter) persisting ≥ 3 days. Follicular diameter were quantified throughout estrus using continuous abdomen ultrasonography, with concurrent measurement of the estrus-to-ovulation interval, Figure 2. Comparative analyses included follicular cyst incidence, ovulation rate, and farrowing outcomes, i.e., total piglets born, born alive, stillbirth rate, mummies rate, total litter weight and piglet index. Data acquisition followed standardized protocols for reproductive parameter assessment in swine models.

### 2.4 Luteinizing hormone (LH) detection

The pre-ovulatory luteinizing hormone (LH) surge serves as the principal endocrine trigger for follicular rupture and ovulation in mammals. To compare ovulation induction efficiency between gonadorelin and buserelin, low-stress blood was collected via caudal venipuncture using needles. Serial blood sampling was performed at 2, 3, 4, 5, 6, and 12 h post-injection. Samples underwent 30-minute clot formation at ambient temperature prior to centrifugation (3,000 × g, 15 min, 4°C). Harvested serum was cryopreserved at −80°C until analysis. LH quantification employed a validated porcine-specific chemiluminescent immunoassay (Beijing Furui Runze Biotechnology, Co., LTD, China) following standardized protocols, with inter- and intra-assay coefficients of variation maintained below 8.0%.

### 2.5 Statistical analysis

The experiment was conducted in three independent replicates. All data are expressed as mean ± SEM and analyzed with SPSS 20.0. Estrus rate, return to estrus rate, pregnancy rate and farrowing rate were analyzed by independent samples *t*-test between the two groups. Chi-square test was chosen for ovulation rate, cumulative ovulation rate and cyst rate analysis. Farrowing index was analyzed by paired samples *t*-test. Additionally, ^*^p < 0.05, ^**^p < 0.01, ^***^p < 0.001. Besides, ^ab^means within the same row with different superscript letters are significantly different (*p* < 0.05).

## 3 Results

### 3.1 Buserelin shortens the estrus-to-ovulation interval in gilts

To compare the estrus-to-ovulation interval following the administration of gonadorelin and buserelin, high-frequency follicular monitoring via abdomen ultrasonography at 8-h intervals was conducted to detect the follicular dynamics and timing of ovulation. Quantitative analysis revealed that no any difference can be detected in follicle size at either GnRH analogs injection or before induced ovulation. Gilts in both groups exhibited an comparable ovulation rate over 95%, as well as a relatively low follicular cyst rate, showing an efficient ovulation induction and considerable safety of both analogs. Importantly, we noticed that the eCG injection-to-ovulation interval tended to be shorter after buserelin administration, in comparison with that of gonadorelin. Further analyses showed that buserelin administration significantly shortened the estrus-to-ovulation interval (*p* < 0.05) (Table 2).

**Table 2:** Follicular dynamics and timing of ovulation in gilts treated with gonadorelin or buserelin.

**Item**	**Gonadorelin**	**Buserelin**
Gilts (*n*)	27	27
Follicle size at GnRH analogs injection (mm)	5.57 ± 0.10	5.51 ± 0.20
Follicle size before ovulation (mm)	6.83 ± 0.20	6.94 ± 0.12
eCG injection-to-ovulation interval (h)	134.73 ± 3.99	129.11 ± 3.55
Estrus-to-ovulation interval (h)	47.7 ± 3.29^a^	37.3 ± 1.99^b^
Ovulation rate (%)	95.54 ± 1.77	96.64 ± 2.03
Follicular cycst rate (%)	5.63 ± 0.78	5.41 ± 0.93

Sample size: n = 27 from three independent experiments. ^ab^means within the same row with different superscript letters are significantly different (p < 0.05).

### 3.2 Buserelin-induced ovulation is more centralized

**Figure 3:**
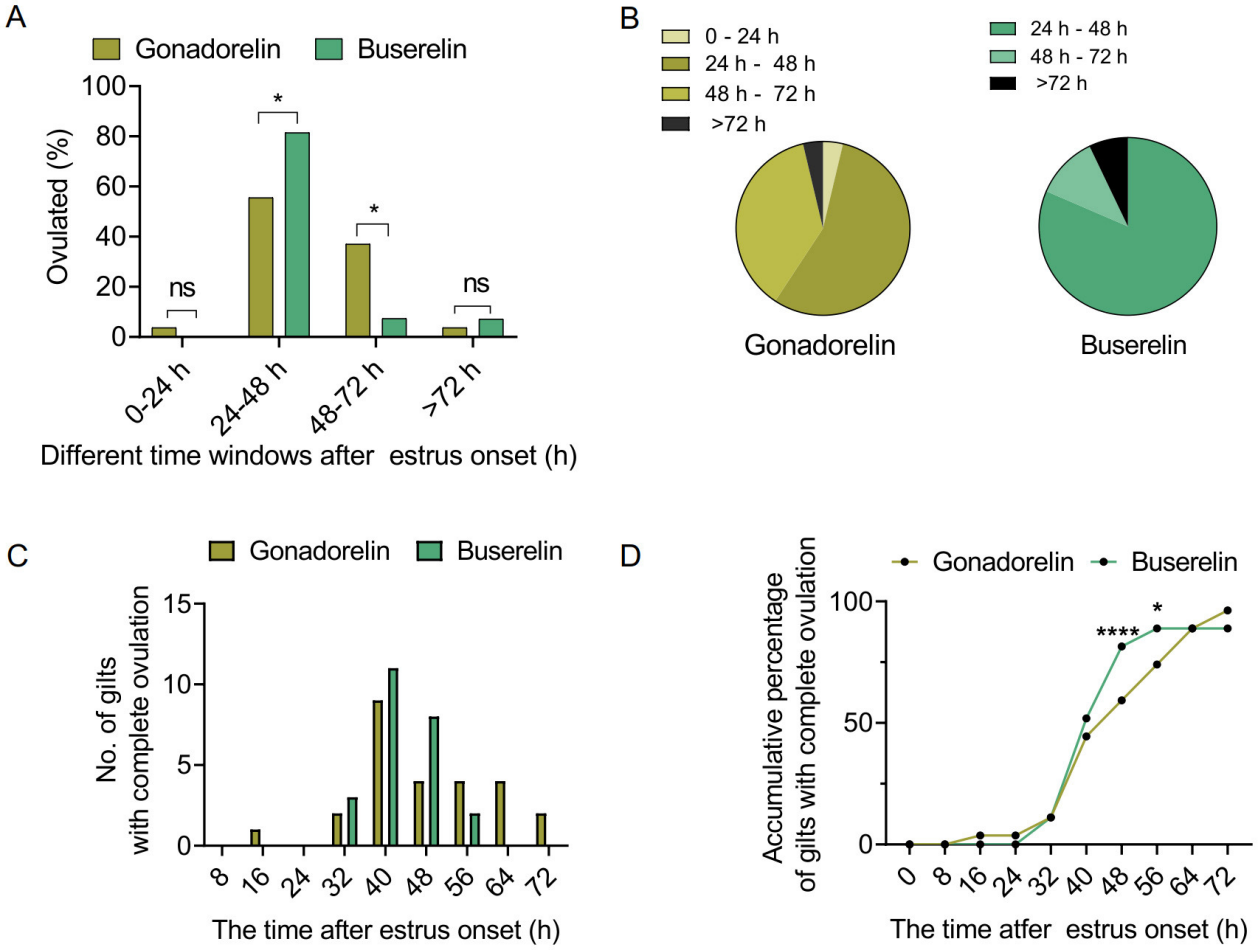
Quantitative analysis of ovulation dynamics. **(A)** Percentage of gilts that have ovulated at different time windows in response to gonadorelin or buserelin. **(B)** Proportion of sows that ovulated at different time windows in two groups. **(C)** Number of gilts that ovulated at different time points. **(D)** Accumulative percentage of gilts that ovulated at the indicated time points. Data are presented as the mean ± SEM, ns, *p* > 0.05; *p < 0.05, and ****p < 0.0001. Gonadorelin group, *n* = 27; buserelin group, *n* = 27.

To assess degree of the ovulation synchronization induced by gonadorelin or buserelin, more detailed quantitative analysis of follicular ultrasonography were performed. The estrus-to-ovulation interval was significantly shortened in the buserelin treatment group (*p* < 0.05) (Table 2). Buserelin administration induced more centralized ovulation, with 81.5% occurring with the 24–48 h post-injection window, which is significantly higher than that (55.6%) following gonadorelin administration (Figures 3A, B). High-frequency dynamic analyses showed that buserelin-induced ovulation occurred earlier than that induced by gonadorelin (Figure 3C). Cumulative analysis further indicated that at 48 h after buserelin injection, over 80.0% of sows have ovulated. In contrast, at the same time point, < 60% of sows treated with gonadorelin have ovulated (Figure 3D).

### 3.3 Buserelin induces a single and centralized LH surge

**Figure 4:**
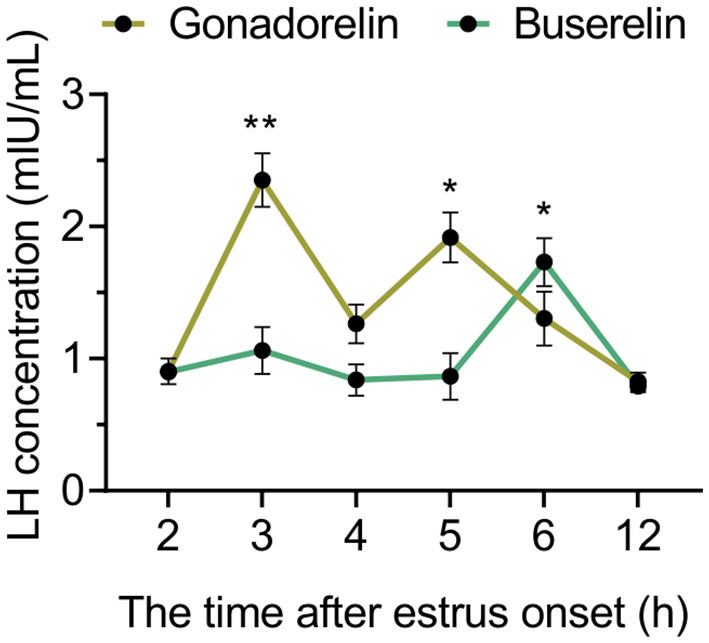
The dynamic of LH concentrations. After gonadorelin and buserelin injection, blood was collected and LH level were analyzed compared to the value of 2 h time point of their own group. Data are presented as the mean ± SEM, *p < 0.05, and **p < 0.001. Gonadorelin group, *n* = 4; buserelin group, *n* = 4.

To explain the beneficial effect of buserelin on centralizing the ovulation, we detected endogenous LH levels after the injection of two GnRH analogs respectively. The gonadorelin injection induced the LH surges at 3 and 5 h post-injection, while buserelin injection induced a single LH surge at 6 h post-injection (Figure 4).

### 3.4 Buserelin improves pregnancy outcomes after FTAI

**Figure 5:**
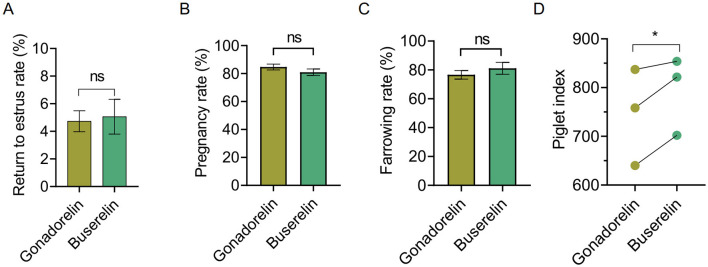
Return to estrus rate, pregnancy outcomes and farrowing performance of gilts treated with gonadorelin and buserelin. **(A)** Return to estrus rate, **(B)** Pregnancy rate, **(C)** Farrowing rate, and **(D)** Piglet index comparison between gonadorelin and buserelin groups using paired two-tailed Student's *t*-test. Data are presented as the mean ± SEM, ns, *p* > 0.05; *p < 0.05. Gonadorelin group, *n* = 120; buserelin group, *n* = 104.

We next compared pregnancy outcomes and farrowing performance in gilts treated with gonadorelin and buserelin. Although the return to estrus rate (Figure 5A), pregnancy rate (Figure 5B), the farrowing rate (Figure 5C) were comparable between two groups. Also no significant difference can be detected in total litter weight, as well as the number of mummies and born alive (Table 3). However, buserelin showed a significant benefit in enhancing following performance, as reveal a higher piglet index (*p* < 0.05) (Figure 5D).

**Table 3:** Farrowing performance of gilts treated with gonadorelin and buserelin.

**Item**	**Gonadorelin**	**Buserelin**
Gilts (n)	120	104
Total piglets born (n)	12.71 ± 0.30	13.89 ± 0.24
Born alive (n)	11.63 ± 0.24	11.55 ± 0.40
Stillbirth rate (%)	1.65 ± 0.15^a^	0.74 ± 0.15^b^
Mummies (%)	0.60 ± 0.11	0.47 ± 0.14
Total litter weight (kg)	13.93 ± 0.30	12.95 ± 0.49

A total of 120 samples were allocated to gonadorelin and 104 to buserelin, sourced from three independent trails. ^ab^means within the same row with different superscript letters are significantly different (*p* < 0.05).

## 4 Discussion

Optimizing the timing of ovulation is crucial for enhancing reproductive performance in pig reproduction management (11, 25). It has been documented that a reduction in the estrus-to-ovulation interval, facilitates fertilization and benefits pregnancy outcomes after the FTAI procedure and is accompanied by an increase in mean piglet birth weight (14, 34). However, the estrus-to-ovulation interval in traditional FTAI protocols is poorly understood (29, 32). By directly comparing two widely used GnRH analogs, we showed that buserelin injection induced an earlier and more centralized ovulation, achieving shorter estrus-to-ovulation interval. The present study supported a tight temporal association between the LH surge and the onset of estrus, which aligns well with findings from other research reporting that LH peaks consistently appear within 12 h after estrus (35). In this study, the interval from estrus onset to the LH peak following gonadorelin treatment was ~3 h, closely resembling the natural interval observed in sows during spontaneous estrus (36). Due to the lack of additional LH data collected at high frequency for reference, we hypothesize that the extended interval between estrus onset and the LH peak observed in the buserelin-treated group may better support the cytoplasmic maturation of the oocyte and quality maintenance under induced ovulation conditions, thereby potentially enhancing oocyte quality (37–39).

Although the estrus-to-ovulation interval could be shortened to about 40 h in gilts induced by hCG (31, 34), the current protocols for inducing ovulation synchronization in FTAI procedures, are mainly based on the use of GnRH (2) and its analogs, such as gonadorelin (16), buserelin (7), triptorelin (40), peforelin (41) and maprelin (42). While various ovulation-inducing drugs were routinely implemented in swine production, pharmaceutical costs remained a critical consideration alongside efforts to enhance sow reproductive performance. Ellen de Jong reported that gonadorelin treatment could improve gilt reproductive performance with an increase of 1.3 total-born piglets per litter (42). Besides, gonadorelin demonstrated a significant advantage over maprelin in reproductive performance, with an average increase of ~1.8 total-born piglets per litter (42). While both ovulation-inducing analogs exhibited comparable cost profiles in the present study, buserelin demonstrated centralized ovulation potential, with experimental piglet index revealing a mean increase of 0.99 live-born piglets per sow, underscoring its broader applicability for optimizing swine reproductive efficiency. However, the estrus-to-ovulation interval has not been directly compared between gonadorelin and buserelin. This is practically of significance not only for improving subsequent pregnancy outcomes, but also, more importantly, for developing single insemination (27). Dispersed ovulation may undermine synchronization thus impeding subsequent pregnancy and farrowing performance (5, 43, 44). In line with the shorter estrus-to-ovulation interval and more centralized ovulation, our study presented improved outcomes after buserelin injection, despite the variations after buserelin administration in the previous study (11).

Even using the FTAI procedure, our previous study has reported that an accurate ovulation detection is beneficial for subsequent farrowing rate and piglet index (15). Thus, we used this optimized FTAI procedure, in which gilts received gonadorelin or buserelin at estrus onset. Though the administration of buserelin did not yield show a statistically significant increase in total little born compared to gonadorelin. We hold the opinion that these unexpected results may be attributed to suboptimal detection sensitivity during early estrus manifestation, leading to temporal misalignment in ovulatory synchronization protocols with GnRH analogs. Notable, within individual batches, buserelin administration demonstrated statistically significant improvements in litter size. Besides, our results further support the advantage of optimizing the FTAI procedure when using buserelin, and emphasize the importance of centralizing ovulation for the FTAI procedure. Thus, integrating estrus detection with buserelin administration may offer a promising strategy for improving the reproductive performance of gilts in the FTAI procedure.

## 5 Conclusion

In direct comparison with gonadorelin, buserelin shows advantages in centralizing induced ovulation and thus improving FTAI outcomes. Therefore, buserelin may be a promising ovulation-inducing drug for optimizing the current gilt FTAI procedure.

## Data Availability

The original contributions presented in the study are included in the article/supplementary material, further inquiries can be directed to the corresponding author.
